# Multifunctional Sensor Array for User Interaction Based on Dielectric Elastomers with Sputtered Metal Electrodes

**DOI:** 10.3390/ma17235993

**Published:** 2024-12-06

**Authors:** Sebastian Gratz-Kelly, Mario Cerino, Daniel Philippi, Dirk Göttel, Sophie Nalbach, Jonas Hubertus, Günter Schultes, John Heppe, Paul Motzki

**Affiliations:** 1 Smart Material Systems, ZeMA—Center for Mechatronics and Automation Technology, 66121 Saarbruecken, Germany; dirk.goettel@htwsaar.de (D.G.); sophie.nalbach@uni-saarland.de (S.N.); paul.motzki@zema.de (P.M.); 2 Sensors and Thin Film Group, University of Applied Sciences, 66117 Saarbruecken, Germany; mario.cerino@imsl.uni-saarland.de (M.C.); jonas.hubertus@htwsaar.de (J.H.); guenter.schultes@htwsaar.de (G.S.); john.heppe@htwsaar.de (J.H.); 3 Department of Systems Engineering, Saarland University, 66123 Saarbruecken, Germany; s8daphil@uni-saarland.de

**Keywords:** dielectric elastomer sensing array, thin-film electrodes, crimped dielectric elastomer, textile-integrated sensor, human–machine interaction, pattern recognition, dielectric elastomers

## Abstract

The integration of textile-based sensing and actuation elements has become increasingly important across various fields, driven by the growing demand for smart textiles in healthcare, sports, and wearable electronics. This paper presents the development of a small, smart dielectric elastomer (DE)-based sensing array designed for user control input in applications such as human–machine interaction, virtual object manipulation, and robotics. DE-based sensors are ideal for textile integration due to their flexibility, lightweight nature, and ability to seamlessly conform to surfaces without compromising comfort. By embedding these sensors into textiles, continuous user interaction can be achieved, providing a more intuitive and unobtrusive user experience. The design of this DE array draws inspiration from a flexible and wearable version of a touchpad, which can be incorporated into clothing or accessories. Integrated advanced machine learning algorithms enhance the sensing system by improving resolution and enabling pattern recognition, reaching a prediction performance of at least 80. Additionally, the array’s electrodes are fabricated using a novel sputtering technique for low resistance as well as high geometric flexibility and size reducibility. A new crimping method is also introduced to ensure a reliable connection between the sensing array and the custom electronics. The advantages of the presented design, data evaluation, and manufacturing process comprise a reduced structure size, the flexible adaptability of the system to the respective application, reliable pattern recognition, reduced sensor and line resistance, the adaptability of mechanical force sensitivity, and the integration of electronics. This research highlights the potential for innovative, highly integrated textile-based sensors in various practical applications.

## 1. Introduction

The use of textile-integrated sensing and actuation elements is important in different application fields and use case scenarios. In recent years, the demand for smart textiles has surged across various sectors, including healthcare [1,2], support of elderly individuals [3], sports and fitness [4], industry, and wearable electronics [5], driven by advancements in flexible and wearable technology [6,7]. The further development of suitable and highly integrated input-sensing elements for user interaction can expand the field of smart textiles. Among the various sensing technologies, dielectric elastomer (DE)-based sensors stand out for their flexibility, lightweight nature, and compatibility with textile integration [8,9]. DE-based sensors can be used for strain and pressure sensing as well as motion detection in wearable systems [10,11,12,13]. When embedded into textiles, DE-based sensors can adapt seamlessly to the body or external surfaces, offering continuous sensing without compromising user comfort. The versatility of DEs allows them to be integrated into a wide range of fabrics, opening doors for innovative applications in both everyday and specialized settings. Sputtered metal-based thin-film electrodes for DEs offer further advantages for textile-integrated elements. The sputtering process and the subsequent laser structuring enable very small structure sizes of the electrodes (sub-millimeter range) [14]. Additionally, the resistance of the electrodes is reduced by orders of magnitude compared to standard carbon-based materials, which is an advantage for sensor applications. The main drawback of using metal-based thin-film electrodes, namely their lack of elasticity, can be overcome by applying corrugated [15] or wrinkled elastomer surfaces [16].

Besides their sensing capability, DEs may simultaneously provide mechanical actuation. By using this ability in a later state of the presented matrix, a complex multifunctional interaction element for user interaction can be provided.

The following contribution describes the development of a small, smart sensing array to sense the user control input in order to interact with components like robots or machines and virtual objects, for example. The basic idea is to develop a flexible, wearable, and unobtrusive touchpad, which can be worn directly on the wristband or other textiles on the body. By including machine learning algorithms in the sensing system, a higher sensing resolution and better pattern recognition capability are obtained. The DE array is built with a new, recently developed sputtering technique to produce electrodes with very low resistance and small geometries. Additionally, a new method of connecting the DE array to the custom-made sensing electronics via crimping has evolved to guarantee a reliable and safe electrical connection. With the developed system, a self-standing sensing unit is achieved to advance human–machine interaction and to create added value in many other areas, such as sports, medicine, industry, entertainment, and rehabilitation, as well as VR and AR, for example.

In the literature, several two-dimensional touchpads are presented. For example, Ref. [17] presents a two-dimensional textile-based capacitive touchpad with 30 conductive pads. Similarly, Ref. [18] uses combined capacitor fibers as fully woven touchpads, while [19] explores radiofrequency identification (RFID) technology. In [20], a high-resolution fabric touchpad for use with a stylus is developed. In [21], optical fibers are used to produce a 3 × 3 array with multi-touch force sensing capability. Heller et al. [22] integrate flexible touch-sensitive fabrics as touchpads on clothes and prove their functionality.

For wearable touchpads, providing feedback to the user can be important for an efficient user interaction. This is achieved in multiple ways in the literature: Ref. [23] explains the possibility of integrating different kinds of actuators into textiles. For example, Ref. [24] uses electroluminescent displays to stimulate visual feedback and, in [25], an array-structured loudspeaker is developed. In most cases, tactile feedback elements are used by combining them with array-shaped sensing units. Different types and structures of wearable haptic feedback elements are compared in [26]. Specifically for tactile feedback elements, DE technology is eminently practicable: for example, Ref. [27] uses dielectric elastomers in an array with tactile feedback. Additionally, Refs. [28,29] show dielectric elastomer-based haptic displays. DEs have the advantage of simultaneous sensing and actuation capabilities [30,31,32], which can be an improvement to wearable and textile-integrated user interaction elements. Furthermore, Ref. [33] shows that DEs can be used for multifunctional feedback, including simultaneous tactile and acoustic feedback.

This paper describes the functional principle of DE sensors and the design of the sensing array. The manufacturing process of the sputtered DE sensor array is explained and includes laser structuring of the electrode, crimping for electrical connections, working principle and design of the electronics, as well as the overall system structure. The results, including single measurements and the performance of pattern recognition algorithms, are discussed afterwards.

## 2. Materials and Methods

In the literature, three different working principles of textile-integrated physical stretch sensors are considered. Either resistive, capacitive (DEA technology), or triboelectric sensing technology has been utilized [34]. Elastic silicone-based capacitive sensors enable the recording of movement, body posture, and pressure distribution over a long wearing period. The combination of textiles and flexible sensors creates integrated measurement systems that are comfortable to wear while individually adapted to the shape of the body. Comfortable long-term measurements can therefore be used for diagnostic and therapeutic support of patients but also for sports applications or in ergonomics.

Silicone offers high stretchability, extremely high chemical and mechanical robustness, and is ideal for integration into stretchable textiles. A modification of the silicone composition permits a wide adjustment range of the modulus of elasticity and, thus, an adaptation to the softness of the textile. Silicone can be used to attach sensors directly to textiles [35], which makes silicone-based DEs a suitable technology for these sensing applications. Due to the specific material properties, such as mechanical flexibility, high alternating load resistance under tension (more than 1 million cycles at 100% elongation), thin structure size (thickness 20–100 µm), high thermal endurance (−40 to 180 °C), and miniaturization capability, silicone-based DEs are optimal for textile-integrated senor elements.

In this chapter, a 3 × 3 DE sensor array for textile integration is developed and validated. The DE array is based on a sputtered metal electrode to enable small electrode structures. A custom-made, low-cost, real-time evaluation electronic with integrated multiplexers is developed to read the specific sensing values. Machine learning algorithms are used to recognize certain input patterns provided by a user. The proposed sensor array uses a newly invented laser structuring method to shape the metal-sputtered DE electrodes [14] and produce highly flexible, low-resistive capacitive sensors. The electrode resistance is on the order of 400 Ω/square and the electrodes can withstand elongations up to 200% for some configurations [14]. In addition to the recently developed electrode structuring, a crimping process and semi-automated handling aperture to manufacture the DE array elements are developed. 

### 2.1. Functional Principle of Dielectric Elastomer Sensors

**Figure 1:**
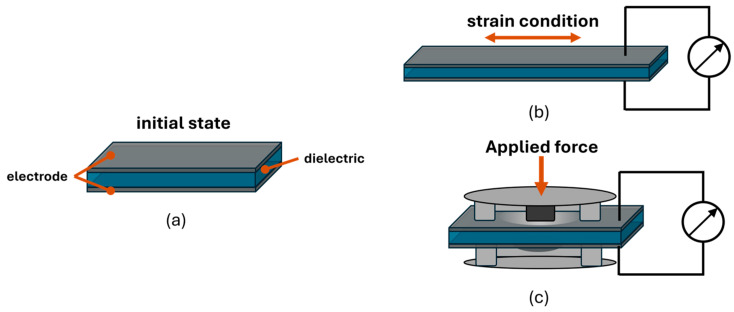
(**a**) Functional principle of DES as (**b**) strain and (**c**) force sensors.

Silicone-based dielectric elastomer sensors (DES) have several advantages for wearable and textile-integrated applications. The softness, light weight, and stretchability of the DES ensure their suitability for embedding into textiles and enable real-time monitoring of body movements, posture, or pressure distribution, without compromising comfort. DES operate via the principle of detecting changes in capacitance caused by mechanical deformation. In textile applications, for example, this sensor typically consists of a flexible dielectric elastomer, sandwiched between two stretchable electrodes (cf. Figure 1) and laminated into the textile. As the textile stretches, compresses, or bends, the shape and distance between the electrodes change, resulting in modification of the capacitance.

This change in capacitance is measured and interpreted by an external circuit to detect mechanical deformations caused by pressure, strain, or motion. For movement and posture sensing, the DES structure can be directly used as stretch sensors. To realize force sensors, in most cases the applied force is transformed into a specific deformation state of the DE membrane to increase the sensor accuracy and adapt to specific force ranges. In Figure 1, the two different functional principles of stretch and force sensors are shown.

To realize a sensing array, the force transformation is performed by a silicone structure on both sides of the DES electrodes (compare Figure 1c). The electrode material needs to be flexible and conductive to enable the functionality of the DES. Conventional electrode materials for DEs are carbon-based materials (carbon black, graphene, and carbon nanotubes), combined with a flexible (silicone-based) carrier substrate. To decrease the electrode resistance and the structure size of the DE element, it is possible to use metal-based electrodes instead of carbon-based electrodes. With the process described in [14], it is possible to manufacture flexible, sputtered, metal-based DE elements.

### 2.2. System Design of the DES Array

**Figure 2:**
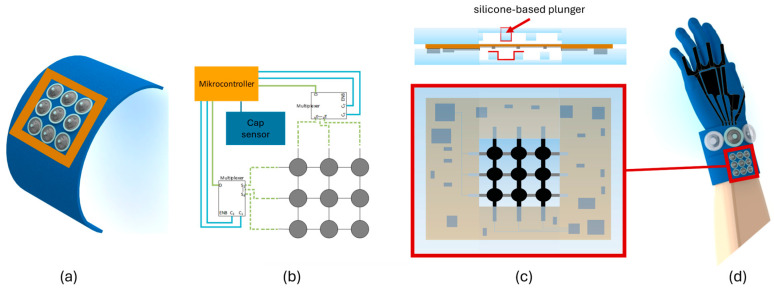
Structure and construction of the sensing array with (**a**) array design and textile integration idea; (**b**) schematic of electronics; (**c**) array and electronics layout with mechanical integration into silicone housing; and (**d**) perspective system integration with additional textile integrated elements.

The presented sensing array consists of 9 sensing elements, with a size of 8 mm × 8 mm each. The deformation of the DE is induced by a silicone-based plunger pressed into the membrane (compare Figure 2c). One silicone frame is attached on each side of the DE membrane with an intersecting structure to increase the resolution of the sensing element. The 3 × 3 array has overall dimensions of 55 × 55 mm, including paths and crimp connectors (see Figure 2). By reading out the capacitance of every single DE element, it is possible to detect where a pressure is applied onto the sensor field. The single sensor lines (rows and columns of the array) are selected using a multiplexer. The specific capacitance of each sensing element is measured by a customized sensing electronic. 

In Figure 2, the configuration of the sensor array is shown, including the electronics layout and the mechanical assembly with silicone frames, which can be bonded to the textile. The electronic components can be arranged around the sensing array and embedded into the silicone housing to form a fully integrated functional unit.

The sensing array can be integrated and combined with further textile-based assistance tools to develop higher level user-interaction units. For example, the system can consist of several sensor elements based on DEs, such as the proposed sensor array or an intelligent sensor glove, with motion and force sensors (compare [13]). In addition, tactile and auditory feedback elements can be used, as shown in the previously developed functional unit [33]. The entire system thus forms a multifunctional user-interaction unit.

### 2.3. Manufacturing Process of the Sputtered DES Array

**Figure 3:**
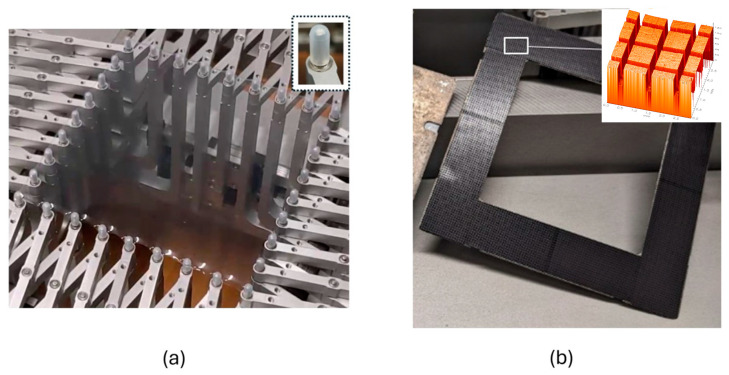
(**a**) DE membrane stretched by use of a custom-made stretching machine. Silicone caps inhibit cracks of the DE membrane and ensure good adhesion during the stretching process; (**b**) one side of the modified carrier system with a magnetic foil on top of a metal frame equipped with a channel system (200 µm width, 60 µm deep); upper right image of the magnetic foil topography captured by means of Chromatic White Light Sensor (Fa. FRT, MicroProf200, Bergisch-Gladbach, Germany).

To manufacture a capacitance sensor array with metal thin films as electrode material, it is necessary to pre-stretch the DE membrane before sputtering the metal thin film on the top and the bottom side of the silicone film. The procedure is described in [16] and produces wrinkles on the surface of the DE membrane after relaxation. Thanks to the wrinkles, it is possible to stretch the sensor array at least to the level of pre-stretch, without damaging the thin-film electrodes. In this work, nickel is used as thin-film electrode material [16]. A modified carrier system is needed to ensure a damage-free processing. Good adhesion on the carrier as well as a damage-free detach of the sputtered DE membrane from the carrier are necessary. In addition, a reliable relaxation process of the coated and pre-stretched DE membrane is important to generate a wrinkle structure on the surface of the nickel-coated DE membrane. Therefore, a metal frame is equipped with a magnetic foil. The magnetic foil (Permaflex 518, Fa. Rheinmagnet Horst Baermann GmbH, Neunkirchen-Seelscheid, Germany), with a thickness of 0.4 mm and a magnetic pressure of approximately 0.2 N/cm^2^, is placed on the top of the metal frame. The custom-made membrane stretcher and the carrier system with the structured magnetic foil are shown in Figure 3.

**Figure 4:**
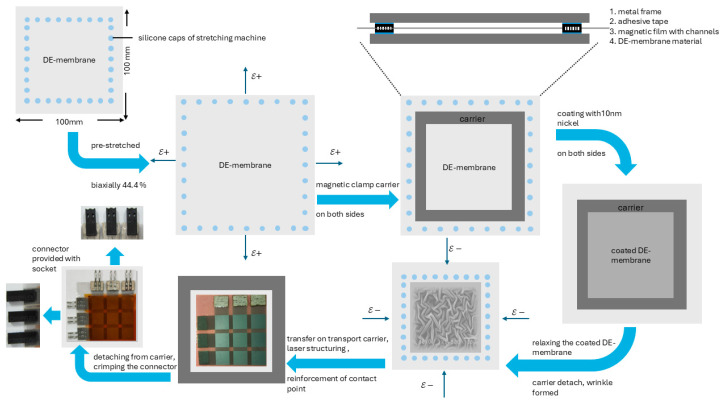
Process steps for manufacturing the thin-film sensor array, starting with attaching the DE membrane on the silicone caps of the stretch machine. Biaxial pre-stretch of the DE membrane to 44.4% and clamping of the DE membrane to the magnetic carrier, followed by coating with 10 nm nickel on both sides. The relaxation process on the stretching machine is supported by isopropanol, while the entire structuring process is realized in three subsequentially structuring steps. Reinforcements of the contact points by hot-melt adhesive fleece, crimping the connectors, and equipment of the female connectors with sockets show the completion of the thin-film-based DE-sensor array preparation. Subsequent encapsulation of the array increases the functionality and improves the integration for different applications.

The process steps to manufacture the thin-film-based sensor array are shown and described in Figure 4. To enable a controllable and reliable relaxation process, the magnetic foil is structured with a micro-channel system for transporting a fluid to support the peeling process. A micro-channel system, 200 µm in width and 60 µm in depth, is structured by a picosecond laser (Fa. 3D-micromac with laser source Fa. Lumera, λ = 355 nm, Chemnitz, Germany) into the magnetic foil. This represents a good compromise between holding force of the silicone DE membrane on the carrier during the sputtering process and during detachment, when isopropanol is used as the liquid to enter the channels by capillary force. 

The manufacturing process of the thin-film metal DE sensor array is divided into four steps, while the generation of the thin-film DE electrodes is described in three subheadings (compare Figure 4):

1. Manufacturing of double-sided sputtered thin-film DE electrode;
2. Laser-based structuring of 3 × 3 sensor array;
3. Electrical interconnection of the sensor array;
4. Encapsulation of the sensor array.

### 2.4. Manufacturing of Double-Sided Sputtered Thin-Film DE Electrode

To realize a uniform and reproducible pre-stretch level of the elastomeric membrane, a custom-made stretching machine is used. The elastomeric membrane material ELASTOSIL 2030 with a nominal thickness of 50 µm ± 5% (Wacker Chemie AG, Munich, Germany, ELASTOSIL^®^ Film 2030) is used for the sensor array presented in this work. The DE membrane can be pre-stretched precisely and reliably, either biaxially or uniaxially. This pre-stretch is essential for the formation of wrinkles and therefore for the entire functionality of the metal thin-film-based DE sensor. Silicone caps on the holding pins of the stretching machine ensure a good adhesion of the DE membrane and inhibit damages during the pre-stretch process (compare Figure 3a).

In this work, all coated DE membranes are initially biaxially pre-stretched to 44.4%. This level of pre-stretch is enough for the thin-film-coated DE membrane to pass all the process steps without any damage and to withstand the mechanical stress during the sensor array application. A 10 cm × 10 cm sheet of the DE membrane is placed on the holding pins of the machine (see Figure 4). Subsequently, the holding pins can move apart automatically. One carrier is placed on the top and another one on the bottom side of the DE membrane. The magnetic force clamps the DE membrane between the two carrier sides and holds the pre-stretch level. The carrier, equipped with the DE membrane, is placed in the sputtering chamber for depositing the nickel electrodes.

For the deposition of the nickel thin film as an electrode material, a sputter vacuum chamber (Fa. CCR) is used [16]. A thickness of approximately 10 nm nickel ensures a good conductivity of about 2 magnitudes lower than conventional carbon black electrodes [16]. The double-sided sputtered DE membrane is placed on the pins of the stretching machine. By dropping isopropanol on the inner border of the carrier, the micro-channel fills with isopropanol and the entire carrier can be detached easily from the DE membrane. The pins move back to the initial position to relax the coated DE membrane, obtaining a wrinkled surface. In the following, the DE membrane is clamped again onto a carrier without pre-stretch for a better handling to the next process step.

### 2.5. Laser-Based Structuring of 3 × 3 Sensor Array

**Figure 5:**
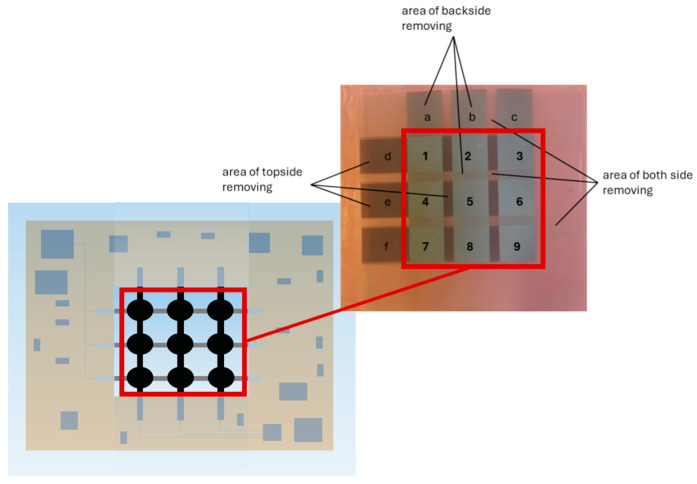
Thin-film-based sensor array; (1)–(9) capacitive sensing areas (structured by UV-picosecond laser); (a)–(c) topside contact points for electrical connection of topside electrodes; (d)–(f) backside contact point for electrical connection of the backside electrodes. As an example, (a) is contacting the topside electrode of the capacities (1), (4), and (7); and (d) is contacting the backside electrodes of the capacities (1), (2), and (3). Thus, all capacitances are electrically connected, and their changes can be detected.

**Figure 6:**
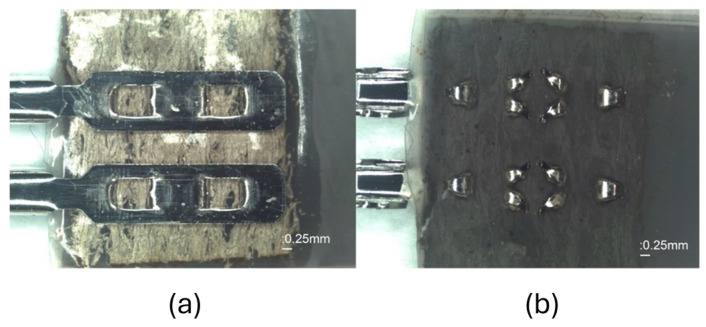
(**a**) Topside of one contact point of the DE membrane sensor array equipped with thin film, conductive hot-melt fleece material, and the two female crimp connectors; (**b**) backside of one contact point; the female crimp connector is pierced through the thin-film DE membrane with the conductive hot-melt fleece.

**Figure 7:**
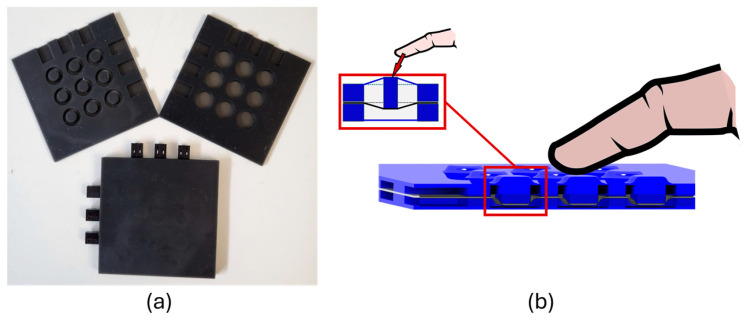
Structure of the sensing array silicone housing with preload pistons to enable a force measurement; (**a**) picture of the housing (top and bottom parts and glued assembled array with crimped contacts); (**b**) schematic and CAD model of the assembled sensing array and a single sensing element.

**Figure 8:**
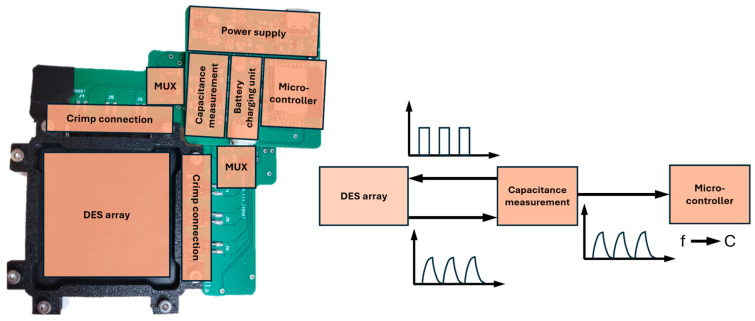
Structure and working principle of the sensing electronics including DES array with silicone housing, crimp connections multiplexers capacitance measurement unit, microcontroller, and power supply with battery charging unit.

**Figure 9:**
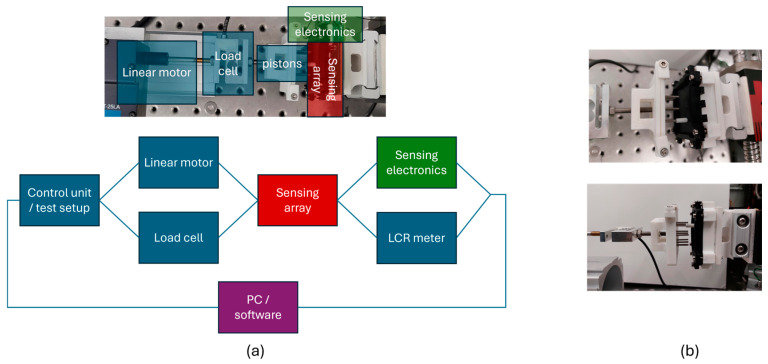
Test setup containing (**a**) test rig structure, linear motor, load cell, and sensing electronics and (**b**) pictures with the sensing array and piston.

The 3 × 3 sensor-array electrode structure is generated by partial ablation of the fully coated DE membrane [14]. At first, the thin film is partially removed only from the topside of the DE membrane. Then, the DE membrane is turned around and the backside is structured. In the last step, the thin film from both sides is removed simultaneously (applying different laser parameters) to separate the contact points and to obtain a measurable sensor array without any short-circuited areas. Thus9 nine capacitors and 6 contact points (see Figure 5, (1)–(9), respectively, (a)–(f)) are ablated from the initially fully coated DE membrane. Every capacitor has an area of 8 mm × 8 mm, while the contact points have 8 mm × 11 mm size.

### 2.6. Electrical Interconnection of the Sensor Array

Due to the very flexible and soft DE membrane material, a good and reliable electrical connection of the thin-film electrodes to the electronic circuit is necessary. Conventional electrical contact methods, like hand soldering of the nickel film, conductive glues, and copper tapes, were tested. However, these methods either lead to a stiffening of the contact points or to an electrical connection that is not sufficiently durable. Investigations of contacting methods were carried out to achieve a reliable electrical contact. The best result is obtained by using a manual crimping press for crimpflex connectors (Fa. Nicomatic, Bons-en-Chablais, France) in combination with a conductive hot-melt adhesive fleece material (e-Web 140, Fa. Imbut GmbH, Greiz, Germany) (see Figure 6).

A lamination temperature of 130–140 °C with a pressure of 0.6 ± 0.04 bar is carried out to connect the conductive hot-melt adhesive fleece material on the contact points of the thin-film sensor array. This ensures an electrical contact with mechanical reinforcement of the contact pads, without over-stiffening before piercing the connector to the contact point. This method creates a reliable connection of the thin-film electrodes without other conductive material. The female connectors are provided with sockets in order to use male connectors for the connection to the electronics.

### 2.7. Encapsulation of the Sensor Array

The structured sensing element, including the crimped connection, is encapsulated in a silicone-based housing to protect the electrodes and the connection against environmental and mechanical influences. Due to the design of the silicone elements, force measurement at each individual measuring position is enabled (compare Figure 1—force sensing principle). The silicone housing is printed with a 3D printer (formlabs—material silicone 40A resin) in two parts, which are bonded on the top and bottom of the DE membrane with silicone glue (SIL-Poxy silicone glue—KauPo, Spaichingen, Germany). The silicone housing includes indentations for the crimps of the electrical connections. The crimp connectors are glued to the housing to ensure mechanical stability of the connections. In Figure 7, pictures and a schematic of the housing are presented.

### 2.8. Design of the Sensing Electronics

The manufactured sensor array is eventually connected to a customized sensor electronic. The main sensing unit is based on commercially available components, performing a cycling charging and discharging time measurement. The DE elements are charged and discharged over a loading and an unloading resistor, respectively. By multiplexing the different columns and rows of the array, the capacitance of the single elements can be measured. The specific DE sensor is cyclically charged to voltage $U_{max}$ and discharged to voltage $U_{min}$ (with $U_{min}<U_{max}<U_{supp}$), and the duration $t_{r}$ of the resulting cycle time, as a sum of charging time $t_{c}$ and discharging time $t_{d\;}$, $t_{r}=t_{c}+t_{d\;}$, is measured. By varying geometry and thickness due to the deformation of the DE, induced by the silicone piston, the capacitance of the DE changes. The capacitance change inevitably results in a change to the overall resulting time $t_{r}$ for the entire charge–discharge cycle. The resulting time is calculated considering the loading and unloading of the resistor, the maximum and minimum voltage, and the capacitance of the DE $C_{DE}$:

(1) $$t_{r}=\left[{R_{1}\ln\left(\frac{U_{supp}-U_{min}\;}{U_{supp}-U_{max}}\right)+R}_{2}\ln\left(\frac{U_{max}\;}{U_{min}}\right)\right]C_{DE}=k\cdot C_{DE}.$$

For an ideal capacitance, the resulting time is directly proportional to the capacitance value. But, considering the DE as a cascade of many resistors and capacities, the behavior is not directly proportional and depends on the internal board frequency (determined by the maximum and minimum trigger voltage). A monotonic relationship between the resulting cycling time and the complex capacitance of the DE is still expected.

Figure 8 shows the designed electronics, including DES array with silicone housing, crimp connections, multiplexers, capacitance measurement unit, microcontroller, and power supply with a battery charging unit. For the prototype, the electronics are realized with standard printed circuit boards (PCB). To integrate the system into textiles, flex-PCB electronics can be used. Additionally, by stacking several electronic boards and optimizing the layout, the electronics’ size can be further miniaturized. For the capacitance measurement unit, a standard bistable flip-flop element is applied. The multiplexers need to have a very low inner parasitic capacitance to enable the measurement of the low capacitive sensing elements.

## 3. Results

The performance of the sensor array is validated in various measurement campaigns to test its applicability for different scenarios. Mainly, capacitance and force measurements are executed to validate the electrical and mechanical behavior of the sensing array.

### 3.1. DES Array Measurement Structure

The measurement structure consists of a linear motor (Aerotech, Fürth, Germany; ANT-25LA) to perform accurate and repetitive measurements by pushing a piston into the sensing array. A load cell (ME-Meßsysteme, Hennigsdorf, Germany; KD40s; 5 N) is connected to the linear axis of the motor to measure the force applied by the measurement piston (simulating the human finger) and validating different force regimes, depending on the silicone housing geometry. In Figure 9, the structure and pictures of the test setup are shown. The sensor array can be mounted in an array holder and a specific sensor element can be selected, with a corresponding holder for the measuring task (see Figure 9b).

The measurement unit consists of the custom-made sensing electronics and an LCR-meter. With the sensor electronics, it is possible to read a single sensor element or to read different sensor elements simultaneously by switching the multiplexer between the different specific connection lines. The motor is controlled via a control unit programed with LabView and the force measurement data are collected with an analog to digital converter, synchronized with the LabView (National Instruments) program. The measured capacitance value is collected on the computer. With a 3D-printed holder for the piston (compare Figure 9b), different elements of the array can be stimulated and different patterns can be applied to the sensor array.

### 3.2. Single Sensing Unit

**Figure 10:**
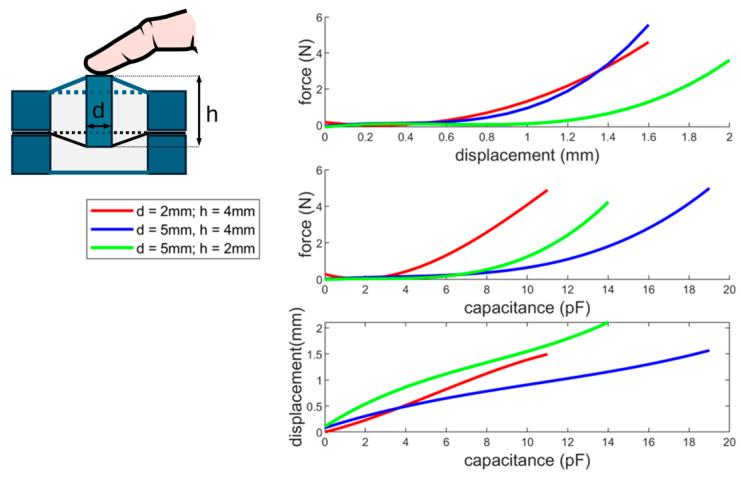
Single element measurements including force–displacement; force–capacitance; and displacement–capacitance measurements for different piston geometries (diameter and height).

The performance of a single sensing unit is characterized to quantify the mechanical and electrical behavior of the single sensor array element. Therefore, the array is manufactured with different geometries of the middle piston (diameter and length). Depending on the geometry of the piston and the hole of the opposite side of the DE membrane, the force behavior of the mechanical system changes. By increasing the diameter of the piston, the feedback force, for pressing the element down, increases. If the height of the piston increases, the DE sensing element is more deformed out of plane in the equilibrium position. This increases on one side the measurement accuracy for slight touches at the equilibrium point but reduces at the same time the maximum deformation range of the element. The user’s subjective tactile sensation is strongly related to the piston geometry. By choosing a certain height of the piston, the individual elements can be felt, which can help to find the specific sensor areas. In Figure 10, characterization measurements for three different piston geometries are shown. With increasing diameter, the force and the capacitance change of the sensing element increases. The force for all three geometries is very low within the first 0.5 mm due to the low membrane stiffness in the equilibrium position. The capacitance change in this region is, nevertheless, in a reasonable region of several pF. The small forces in the low-displacement region can lead to misperceptions of the user and possibly to errors in the data acquisition. To increase the force, it is either necessary to preload the DE stronger in the equilibrium position (which leads to a reduction in the working range) or to change the geometry of the preload element (silicone housing). By reducing the outer diameter and increasing the inner diameter of the silicone housing, the overall force and the force in the low-displacement range increases. Alternatively, the structure may be altered (compare [10,11,13]) to increase the equilibrium force. For the presented case, the simpler geometry is chosen, as this provides sufficient values for the capacitance resolution and, at the same time, fulfills the mechanical properties. The choice of force behavior can vary depending on the application; therefore, an adaptation of the geometry can be useful in other cases. In the presented case, a silicone pad that is flat in the equilibrium position was used (to create the impression of a uniform surface). Therefore, a diameter of 5 mm and a height of 2 mm for the piston is used. The displacement resolution of this geometry is the lowest but, for the application, the resolution is still sufficient.

### 3.3. Array Validation Measurements

**Figure 11:**
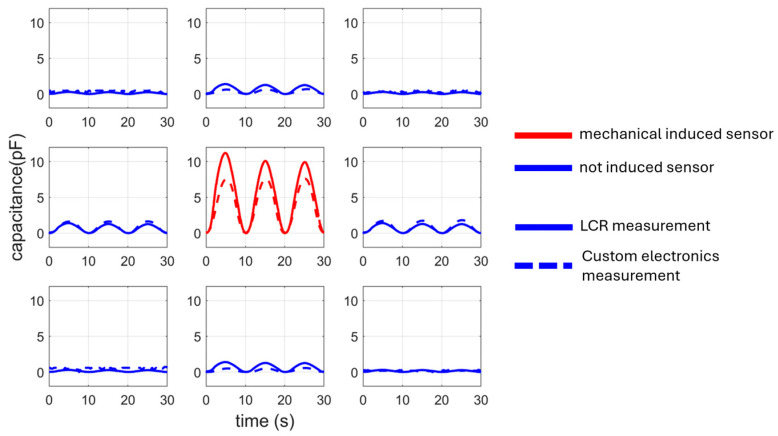
Capacitance measurement comparison for the whole 3 × 3 array with LCR meter vs. custom electronics measurement. The red lines indicate the mechanical stimulated sensor (middle sensor; array position 2 × 2); the blue lines indicate the not mechanically stimulated sensors. The solid lines are the LCR measurements for all sensors and the dashed lines are the sensor electronics measurements switched with the multiplexers.

In Figure 11, two measurements for all elements of the sensing array are presented. In the plots, measurements with the LCR-meter are compared with a measurement using the sensing electronics. For the LCR-meter measurement, one measurement for every array element is performed with the same movement of the motor for the stimulated element (array position 2 × 2). Only one measurement is carried out for the sensor electronics, while the multiplexers switch through the various array elements during the measurement.

### 3.4. Pattern Measurements

**Figure 12:**
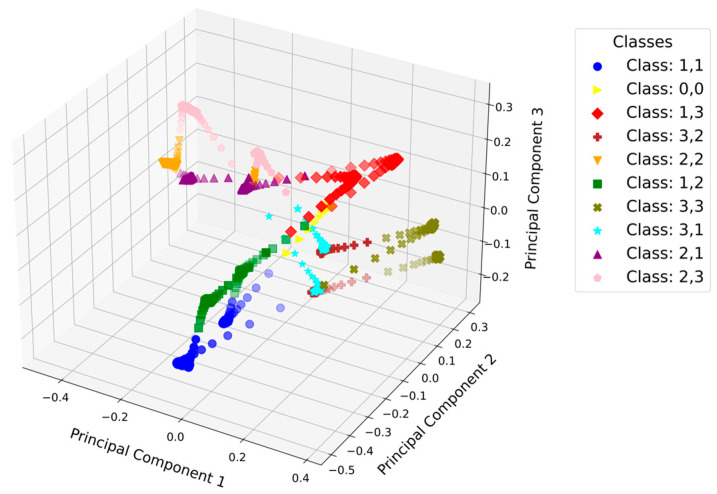
PCA of the training data for the stimulation of every array element (component 1,1 to 3,3 and 0,0 (no element pushed)) with the motor for 1 mm deformation and 2 mm deformation.

The array can be used for more complex input sensing, like control of a robot or text input via touchpads. In Figure 11, the capacitance behavior of the nine elements is shown by pressing the middle array element. To recognize a pattern, the machine learning (ML) algorithm needs to predict the pushed element, depending on the capacitance measurements. Two or more elements may be pressed nearly simultaneously during data collection. This can lead to complicated data processing due to the complex interaction of the single measurements, especially at the unpressed elements. In Figure 12, the principal component analysis (PCA) for the classes of the training data is shown in a 3D plot. The pushing of every element can be assigned to a certain class. In the plot, the difference between pressing the piston 1 mm or 2 mm can be seen as two sets of classes, behaving symmetrically in the 3D space.

For the training of the algorithm, we assume that the user presses the elements after each other (but, under certain circumstances, very quickly). The training is performed by pushing every element to a certain level (1 mm and 2 mm) and labeling the level as a pushed state. The training is performed with a linear motor, moving a piston into the array and holding the deformation for a certain time (compare Figure 9). For every element, this leads to one (e.g., in this case two (1 mm and 2 mm)) label, which can be used in a later measurement to predict the performed pattern.

### 3.5. Pattern Recognition Performance

For the validation of the ML approach, three different patterns are performed on the array and three different algorithms for the pattern recognition (LDA, KNN, and a neural network) are used. The three different patterns are an O-pattern (the outer elements of the 3 × 3 array in a circular movement), an X-pattern, and a Z-pattern. The patterns are performed in different ways; for each of the patterns, the array was stimulated in five different ways:

1. Performed sinusoidal movement pattern of the piston with the linear motor;
2. Pattern performance stimulated by user 1, where the single elements are pushed each after another;
3. Pattern performance stimulated by user 1, where the pattern is introduced in a continuous movement of the finger;
4. Pattern performance stimulated by user 2, where the single elements are pushed each after another;
5. Pattern performance stimulated by user 2, where the pattern is introduced in a continuous movement of the finger.

In Table 1, the prediction performance of the different ML algorithms is shown. In each case, the performance of LDA is higher than that of the other two prediction algorithms. Overall, all LDA-based predictions for the different stimulation variants have an accuracy higher than 80 %, measured for each time stamp of the measurement (~4 Hz time resolution). The K-neighbors algorithm was trained with k = 5 and the neural network consists of an input layer (with nine neurons), one hidden layer (16 neurons (rectified linear unit activation [ReLu]), dropout 40 %), and an output layer with 10 neurons (softmax activation).

Table 1 shows that continuous sweeping, in most cases, has a lower data quality due to possible signal overlaps. This phenomenon can be explained by the pressing behavior of the user. Not every position is held with constant pressure. This results in a variance in the data, which is dependent on the user’s pressure on the positions and in-between areas, where no sensor element is located. However, there are also cases where continuous sweeping performs better than single pressing. This can be explained by the invisibility of the sensor surfaces. When pressing the individual sensors, it can therefore happen that the sensor surface is not hit exactly and the corresponding center of the sensor (palpable by plunger) must be felt. This leads to incorrect sensor data and, consequently, to lower reliability of the algorithm evaluation.

The LDA algorithm shows the best accuracy compared to K-neighbor and the neural network. Since the LDA algorithm works best when the data show linear separability, it can be concluded from the results shown in Table 1 that the separation of the patterns corresponds to a linear separation. This is confirmed by the poorer results of the K-neighbor algorithm. This algorithm performs a nonlinear separation of the classes depending on the number of neighbors and the distance calculation. A better choice of parameters could improve the results of the K-neighbor algorithm.

In addition, the neural network performs slightly worse than the LDA algorithm. This can be explained by the nonoptimal choice of the network parameters. For example, a lower number of neurons could simplify the network. The simplification leads to a better separability of the data, under the assumption that a linear separation is preferable for the data.

**Figure 13:**
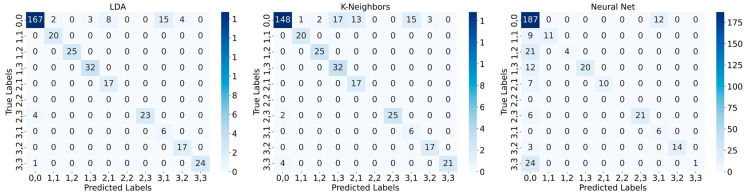
Confusion matrices for the O-pattern, stimulated with the linear motor.

Figure 13 shows an example of the confusion matrices for the O-pattern, induced by the linear motor. During the measurement, most of the entries are labeled as 0,0 (no pattern introduced at this time stamp). All misinterpreted labels were either predicted wrongly to the class 0,0 or the class 0,0 was misinterpreted by the algorithms to another label. In conclusion, all misinterpretations are in the context of 0,0 class labeling. By increasing the accuracy of the algorithms for the not-pressed state (e.g., by reducing the measurement noise or introducing a measurement threshold), the performance of the algorithms can be highly increased.

**Figure 14:**
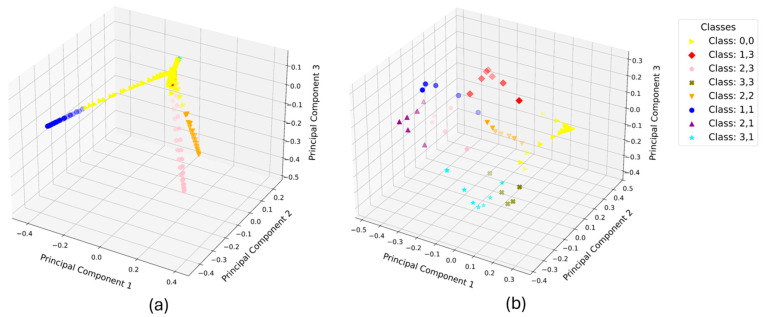
PCA of the measurement data, exemplary for the Z-pattern with (**a**) linear motor compared to (**b**) pushing of the individual elements by user 2.

The PCA of the measurement data is shown in Figure 14 for two measurements of the Z-pattern as an example of the whole measurement campaign. Depicted is the sinusoidal stimulation by the linear motor (Figure 14a) as reference for a repetitive deformation of the sensing elements compared with the stimulation by a user (user 2) pressing the single sensing elements each after another. The ML accuracy of the three different algorithms is very different and varies more for the hand-driven measurements than for the measurement with the linear motor (compare Table 1). The accuracy of the LDA is over 90 % for the user stimulation and even higher than for the motor measurement.

**Figure 15:**
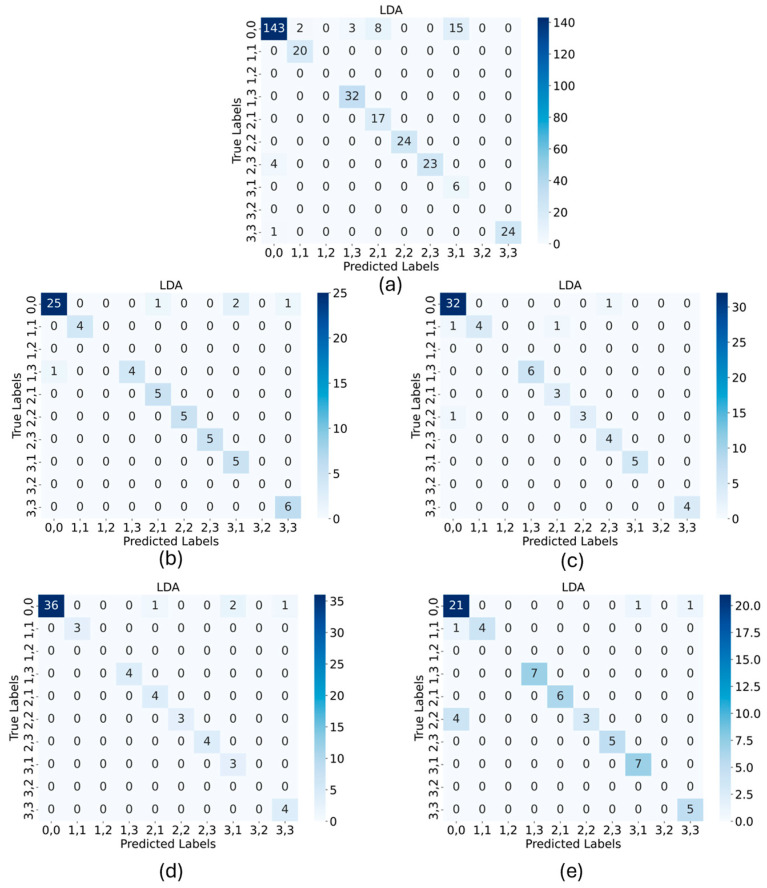
Confusion matrices for the Z-pattern measurements of different stimulation variations: (**a**) linear motor-induced deformation; (**b**) stimulated by user 1 when the single elements are pushed each after another; (**c**) stimulated by user 1 when the pattern is introduced in a continuous movement of the finger; (**d**) stimulated by user 2 where the single elements are pushed each after another; (**e**) stimulated by user 2 when the pattern is pressed as a continuous movement of the finger.

As the performance of the LDA is the highest in all executions, the confusion matrices of the LDA classification for the Z-pattern in all five different stimulation variations are exemplary, shown in Figure 15. Again, for these cases, the misinterpretations of the 0,0 entries are the only misinterpretations of the algorithm.

## 4. Discussion

In the presented paper, a smart sensing array for human–machine and virtual interaction is developed. The design and manufacturing process of the array is documented, while the functionality is validated by different experimental measurement campaigns. For the measurements and the real application, a custom-made capacitance sensor electronic with multiplexers is developed. A microcontroller sends the measurement data wirelessly to the evaluation system. The measurements show that the interaction behavior of the array elements (especially the force deformation behavior) can be shaped by the design of the silicone housing geometry for the individual sensor elements. Additionally, the capacitance behavior of the single sensing elements and the whole array is studied with different measurements. By using various machine learning (ML) algorithms for pattern recognition, the system performance of the array can be validated. The ML algorithms for all performed pattern recognition measurements have a prediction performance of over 80% for LDA, even for the hand-induced measurements.

The system can be further developed and optimized in various ways. One improvement could be the miniaturization of the DE array itself and the sensor electronics in order to achieve a smaller interaction element. The manufacturing process is, with some further development, suitable for volume production. The ML algorithms can further be improved to detect more complex patterns with higher accuracy in real time.

Our contribution shows a three dimensional ‘touch pad’ for textile integration with the ability to measure the induced push intensity. The sensing array is applicable in many different fields like industry manufacturing, entertainment, and human–robot co-operation. By including the actuation capability of DE systems, tactile and even combined audio–tactile feedback can be added to the presented sensing array to reach a higher level of user interaction. Combined with various other user-interaction sensor and feedback elements based on the DE technology, textile-integrated elements can be realized, such as, for example, multi-functional interaction systems. This further expands the application areas of the presented system principal, for example, to sport, rehabilitation, and medicine. In this context, the developed user interface enables the further development of smart textile-integrated user-interaction interfaces for a variety of application fields.

## Figures and Tables

**Table 1:** Prediction performance of the ML algorithm for different patterns and various stimulations of the patterns for the considered algorithms (LDA, KNN, and neural network).

Pattern	Version	LDA	KNN	Neural Net
O	Linear motor	89.9	84.5	74.4
	User 1 continuous sweeping	84.6	69.2	80
	User 1 single pressing	90.8	53.8	69.2
	User 2 continuous sweeping	83.1	70.8	73.8
	User 2 single pressing	86.2	69.2	83.1
X	Linear motor	83.4	82.7	68.6
	User 1 continuous sweeping	92.2	46.9	85.9
	User 1 single pressing	87.5	76.5	76.5
	User 2 continuous sweeping	84.6	53.8	81.5
	User 2 single pressing	89.2	81.5	87.7
Z	Linear motor	89.7	81.4	77.6
	User 1 continuous sweeping	93.8	49.2	84.6
	User 1 single pressing	92.2	67.2	79.7
	User 2 continuous sweeping	89.2	58.5	80
	User 2 single pressing	93.8	47.7	87.7

## Data Availability

The raw data supporting the conclusions of this article will be made available by the authors on request.
